# Supplementary material for: Strain-induced creation and switching of anion vacancy layers in perovskite oxynitrides
Source: Nat Commun. 2020 Nov 23;11:5923. doi: 10.1038/s41467-020-19217-7 (PMC7683707; doi:10.1038/s41467-020-19217-7)
Supplement: Supplementary file 1 — Supprementary Information [file 41467_2020_19217_MOESM1_ESM.pdf]

Supplementary Information

Strain-induced creation and switching of anion vacancy layers in perovskite oxynitrides

T. Yamamoto *et al*.

Contents:

Supplementary Note 1-5
Supplementary Figure 1-10
Supplementary Table 1-6
Supplementary references

Supplementary Notes

Supplementary Note 1. Structural characterization of the powder samples.

We carried out ammonolysis reactions from $SrVO_3$, $SrVO_2H$, and $Sr_2V_2O_7$. Supplementary Fig. 1a shows powder XRD patterns of $SrVO_2H$ samples before and after ammonia treatment. The diffraction pattern of $SrVO_2H$ was indexed in a tetragonal unit cell with a = 3.9347(3) Å and c = 3.6670(4) Å. These parameters are consistent with the reported values.[1] A minor impurity of perovskite $SrVO_{3-\delta}$ (a molar ratio of 4%) was also found. When $SrVO_2H$ was heated with NH_3 at 300 °C, we obtained a cubic perovskite phase with the lattice parameter of a =3.860(1) Å (Supplementary Fig. 1a). The sample color remains black. The full width at half maximum (FWHM) is almost the same as its precursor $SrVO_3$, indicating good crystallinity of the sample. The oxidation of vanadium ions by the ammonolysis reaction is inferred from the similar cell constant with $SrVO_3$. Such an oxidation behavior upon ammonolysis is seen in the titanium oxyhydride perovskite ($BaTi^{3.4+}O_{2.4}H_{0.6}$ → $BaTi^{4+}O_{2.4}N_{0.4}$).[2]

We conducted Rietveld refinement of the synchrotron XRD pattern for the $SrVO_2H$ sample ammonolized at 300 °C using the ideal perovskite structure (space group Pm–3m). An oxygen-deficient phase '$SrVO_{3-\delta}$' was considered because of the negligible X-ray scattering contrast between O and N. We placed Sr, V and O atoms, respectively, at Wyckoff position 1a, 1b and 3c. The refinement was converged reasonably, with the agreement indices of R_p = 5.23%, R_{wp} = 7.27%, R_B = 4.99%, and GoF = 2.32 (Supplementary Fig. 2a and Supplementary Table 1). The oxygen defect concentration was refined to δ = 0.07(2). In order to obtain a N/O distribution at each anionic site, we performed Rietveld refinement using powder neutron diffraction data of the same compound (Supplementary Fig. 2b). The δ value was fixed to the value obtained from synchrotron XRD. The composition was determined as $SrVO_{2.70(4)}N_{0.22(4)}$ and agreement indices are R_p = 3.21%, R_{wp} = 2.88%, R_B = 0.83%, and GoF = 1.50 (Supplementary Table 1). The nitrogen content (2.98 wt%) agrees well with 2.9(3) wt% obtained by the combustion elemental analysis. These results allowed us to set the final composition as $SrVO_{2.7}N_{0.2}$. An increase of the oxygen content from the precursor $SrVO_2H$ may be derived from moisture contamination in the synthetic atmosphere.

The XRD pattern of the $SrVO_2H$ sample ammonized at 600 °C was indexed on a rhombohedral unit cell with a = 5.51 Å and c = 34.3 Å. The impurity phase of VN (16 wt%)[3] was found as well. The sample color stays black. As in the case of the $SrVO_3$-derived sample, the obtained hexagonal pattern resembles with those of the 15R-perovskite $SrCrO_{2.8}$ with a long range oxide-vacancy ordering along the $[111]_p$ direction.[4] Thus, the XRD pattern was refined by using the 15R-type structure (space group R–3m), where Sr1 atoms were placed at 3a (0, 0, 0), Sr2 at 6c (0, 0, z), Sr3 at 6c (0, 0, z), V1 at 6c (0, 0, z), V2 at 6c (0, 0, z), V3 at 3b (0, 0, 1/2), O1 at 6c (0, 0, z), O2 at 18c (x, –x, z), and O3 at 18c (x, –x, z). Again, nitride ions were not considered here. The isotropic displacement parameters U_{iso} of the

same elements were set to be equal. The agreement indices are R_p = 2.48%, R_{wp} = 3.37%, R_B = 1.41%, and GoF = 2.16 (Supplementary Fig. 2e). The full results are given in Supplementary Table 3.

In order to examine the oxygen/nitrogen distribution, we performed Rietveld analysis of neutron diffraction pattern (Supplementary Fig. 2f). We imposed a constraint of $g_{Oi} + g_{Ni} = 1$ for each site (i = 1, 2, 3). The z coordinates and atomic displacement parameters U_{iso} for the vanadium sites were fixed to the value obtained from the X-ray refinement since the scattering length of V is close to zero. The refined occupancy factor of nitrogen atom at the O2 and O3 sites g_{N2} and g_{N3} was ~1/3 and ~1/8, respectively, while no appreciable nitrogen was detected at the O1 site (g_{N1} = –0.04(4)), hence g_{N1} was fixed at zero. Anisotropic displacement parameters U_{ij} were used for O1. The final results are shown in Supplementary Fig. 2f and Supplementary Table 3 (R_p = 3.34%, R_{wp} = 4.29%, R_B = 2.19% and GoF = 1.18). The occupancies of the anion sites are g_{O1} = 1, g_{O2} = 0.66(3) (g_{N2} = 0.34(4)) and g_{O3} = 0.85(3) (g_{N3} = 0.15(3)), thus giving the composition of $SrVO_{2.22(5)}N_{0.58(5)}$. Taking into account the VN impurity (16 wt%), the total nitrogen amounts to 7.20 wt%, in a good agreement with the value obtained from the combustion analysis (7.0(3) wt%).

When $SrVO_3$ was ammonized at 500 °C, we obtained a cubic perovskite phase (Supplementary Fig. 1b). The lattice constant, a = 3.850 Å, is slightly larger than a = 3.843 Å for the oxide precursor $SrVO_3$. Combustion analysis resulted in the nitrogen content of 0.64 wt%, corresponding to $SrVO_{2.85}N_{0.1}$. The vanadium valence remains +4. The same valence is seen in $SrV^{4+}O_{2.7}N_{0.2}$ obtained from $SrVO_2H$ ammonolized at 300 °C. The XRD pattern at the reaction temperature of 600 °C could be indexed by a rhombohedral unit cell with a = 5.51 Å and c = 34.3 Å. It contains an $Sr_3V_2O_8$ impurity phase.[5] The hexagonal pattern obtained also resembles with the $SrVO_2H$ sample after the same ammonolysis treatment. Combustion analysis gave the nitrogen content of 3.3(3) wt%.

We also carried out Rietveld refinement of X-ray and neutron data for the $SrVO_3$ sample nitridized at 600 °C by using the same structural model (see the main text, Supplementary Fig. 2c, 2d and Supplementary Table 2), and obtained in principle the same structure with a composition of $SrVO_{2.203(8)}N_{0.597(8)}$. Taking into account the $Sr_3V_2O_8$ impurity with 16 wt%, the total nitrogen amount in the sample is 3.8 wt%, which is consistent with the value obtained from the combustion analysis (3.3(3) wt%).

Supplementary Note 2. DFT calculation of 15R-$SrVO_{2.2}N_{0.6}$.

We calculated the total energies of all the possible 11 configurations of nitrogen for the supercell consisting of $Sr_5V_5O_{11}N_3$ ($SrVO_{2.2}N_{0.6}$) as a calculation model, where two nitrogen atoms are placed at the O2 site and one nitrogen atom at the O3 site (Supplementary Fig. 3a). This situation (33% and 17% for O2 and O3 sites) approximately corresponds to the experimental ratio of the nitrogen substitution (34% and 15% for O2 and O3 sites). The unit cell vectors were set to be $a_1 = (a/2, -(\sqrt{3}/2)a, c/3)$, $a_2 = (a/2, (\sqrt{3}/2)a, c/3)$, and $a_3 = (-a, 0, c/3)$. We optimized both the lattice constants (the *a* and *c* axes) and atomic coordinates, while the cell shape was kept fixed. The plane-wave cutoff energy of 600 eV and a 6 × 6 × 6 *k*-mesh were used. We found that the most stable structure includes the *cis*-VO_4N_2 octahedra (Supplementary Fig. 3a). The total energy of structure with *trans*-VO_4N_2 octahedra is at least 0.2 eV/f.u. higher than the most stable one. The possible preference of the *cis*-configuration was also suggested in $NdVO_2N$.[6]

By using the most stable configuration of $Sr_5V_5O_{11}N_3$, first-principles band-structure calculation and the subsequent Wannier construction for the V-*d* orbitals were performed to investigate electronic structure. The unit cell vectors were changed in the band-structure calculation by the WIEN2k code as shown in Supplementary Fig. 3a. The partial density of states (pDOS) is presented in Supplementary Fig. 3b. The *RK*max parameter used in the WIEN2k code was set to be 7.0. The octahedral coordinate V2 and V3 sites have significant DOS between −1 to 0 eV while the tetrahedral V1 site has almost no DOS at the same energy region. The electron occupancies are evaluated as 0.2, 1.1, and 1.4 electrons for V1, V2 and V3, respectively. This suggests that the V2 and V3 sites are nearly tetravalent while the V1 site is pentavalent, in consistency with the BVS calculation (Supplementary Table 4) and NMR (Fig. 2). The first-principles band structure and the band structure calculated with the tight-binding model consisting of the Wannier orbitals are shown in Supplementary Fig. 3c. The Fermi surface was calculated with the tight-binding model derived here using a 100 × 100 × 100 *k*-mesh, and depicted using the FermiSurfer[7] as shown in Fig. 3a.

Supplementary Note 3. Characterization of the oxynitride thin films deposited on LSAT (111).

The oxynitride film on LSAT (111) was obtained by ammonolysis reaction of the epitaxially grown $SrVO_3$ (600 °C, 12 hours). EDS spectra implied the formation of oxynitride after ammonolysis since a nitrogen K_α was clearly observed (Supplementary Fig. 4g). The retention of epitaxy in the oxynitride film was confirmed by reciprocal space mapping (Supplementary Fig. 4d, 4e). The 111 peak of the thin film shifts to lower angle by the ammonolysis treatment, which is the same tendency with the ammonolysis of the bulk sample (Supplementary Fig. 4b, 4c). However, no superlattice peak could be detected in out of plane XRD at the low angle, suggesting the vacancy formation of the thin film and bulk are rather different. Figure 4a shows high angle annular dark field scanning transmission electron microscopy (HAADF-STEM) image of the thin film sample. A domain of dark stripes is visible in the film along $(112)_p$, indicating the formation of a superstructure along $(112)_p$. The evidence of superstructure is also found in the Fourier transform (FT) pattern; As shown in the inset in Fig. 4a, superlattice spots appear along $[112]_p$ direction at one-seventh intervals between fundamental spots of the primitive perovskite structure. These results suggests that the anion vacancies are formed along $(112)_p$ with the 7-fold superlattice, in stark contrast to the bulk sample with vacancies along $(111)_p$. In-plane XRD measurement also shows the 7-fold superlattice along $[112]_p$, where $(1/7\ 1/7\ 2/7)_p$ peak is observed at 2θ = 7.8° (d = 11.3 Å; Supplementary Fig. 4h). The equivalent $(2/7\ 1/7\ 1/7)_p$ and $(1/7\ 2/7\ 1/7)_p$ peaks were also observed (Supplementary Fig. 4i, 4j), indicating that the three types of domains are rotated by 120° with respect to each other.

Since the scattering intensity in HAADF imaging is approximately proportional to the square of the atomic number, Sr and V columns appear as the brighter dots in the HAADF image (Supplementary Fig. 4a). In the annular bright-field (ABF) image, anion columns are also visible between cation columns as darker dots (Supplementary Fig. 4a). Displacements of anion columns from $(112)_p$ planes are observed (Fig. 4e), indicating formation of $V(O,N)_4$ tetrahedra around anion vacancies, as found in the 15R-type structure. For the structural model, we assumed a monochromic cell with seven octahedra along the c axis with β = 124° (a white frame in Supplementary Fig. 4a). Then, the lattice parameters are determined as a = 7.11 Å, b = 5.48 Å, c = 27.0 Å from the XRD data shown below:

1) (2 1 –7) (= $(1\ 0\ 1)_p$) : d = 2.80 Å (Supplementary Fig. 4f)
2) (1 0 7) (= $(1\ {-1}\ 0)_p$) : d = 2.32 Å (Supplementary Fig. 4e)
3) (0 2 0) (= $(1\ 1\ 1)_p$) : d = 2.74 Å (Fig. 4a)
4) (0 0 2) (= $(1/7\ 1/7\ 2/7)_p$) : d = 11.2 Å (Supplementary Fig. 4h)

The model structure (7M-structure) with vacancy planes along $(112)_p$ is shown in Supplementary Fig. 4a (structural parameters are shown in Supplementary Table 5). The calculated HAADF and ABF images from the 7M-structure reproduces well the experimental ones (Fig. 4e and Supplementary Fig.

4a). The composition obtained from this model is $SrV(O,N)_{2.71}$ ($Sr_{14}V_{14}(O,N)_{38}$). Nuclear reaction analysis (NRA) and elastic recoil detection analysis (ERDA) gave the nitrogen content of $x = 0.8(2)$ and $x = 0.54(3)$, respectively, in $SrVO_{2.71-x}N_x$. The positions of nitride ions could not be determined by the current experiments, but we deduce that the apical anions of tetrahedra are occupied only by oxygen atoms as in 15R-$SrVO_{2.2}N_{0.6}$. The bond lengths of V–O and Sr–O are fairly consistent with the bond lengths for 15R-$SrVO_{2.2}N_{0.6}$ (Supplementary Tables 4 and 6). Assuming oxide ions for all anion sites, BVS of +3.3~3.5 are obtained for the octahedral and pyramidal vanadium sites, while +4.4 is obtained for the tetrahedral site. It is thus expected that, similarly to 15R-$SrVO_{2.2}N_{0.6}$, the tetrahedral vanadium is V^{5+} and the octahedral and pyramidal vanadium are $V^{3\sim4+}$.

Supplementary Note 4. Characterization of the oxynitride thin films deposited on $LaAlO_3$ (111) and $SrTiO_3$ (111).

The oxynitride film on $LaAlO_3$ (111) was obtained by ammonolysis reaction (620 °C, 12 hours). EDS spectra implied the formation of oxynitride after ammonolysis since the N K_α peak was observed (Supplementary Fig. 5b). The 111 peak of the thin film shifts to lower angle by the ammonolysis. While no superlattice peak could be detected in out of plane XRD at the low angle (Supplementary Fig. 5a), in-plane XRD measurement shows the seven-fold superlattice along $[112]_p$, $[121]_p$ and $[211]_p$ (Supplementary Fig. 5c-5e), suggesting that the structure is identical to that of the film on LSAT.

The oxynitride film on $SrTiO_3$ (111) was obtained by ammonolysis reaction (620 °C, 12 hours). EDS spectra the N K_α peak, implying the formation of oxynitride after ammonolysis (Supplementary Fig. 6c). The 111 peak of the thin film shifts to lower angle by the ammonolysis, the same tendency as the bulk sample (Supplementary Fig. 6a). If one assumes the 15R-structure where the LSAT [111] direction corresponds to the *c* direction of 15R structure, superlattice refractions, such as $(1/5\ 1/5\ 1/5)_p$ ($2\theta \sim 7.7°$), are expected to be observed by out of plane XRD. However, we observed a superlattice peak at 6.5° (Supplementary Fig. 6b) which corresponds to $(1/6\ 1/6/\ 1/6)_p$ reflection, indicating a six-fold superstructure with $(111)_p$ planar vacancy. The six-fold superstructure along $[111]_p$, was also observed in Fourier transform (FT) pattern (inset in Fig. 4b). Figure 4b shows HAADF-STEM image of the thin film sample. A domain of dark stripes is visible in the film along $(111)_p$. These results suggests that the anion vacancies are ordered along $(111)_p$ with the 6-fold superlattice.

Supplementary Note 5. Thermodynamic competition between SVON-111 and -112 as a function of biaxial strain.

To evaluate the preference of the $(111)_p$ and $(112)_p$ anion vacancy orientations of $SrVO_3$ after ammonolysis (SVON), we calculate their relative free energies as a function of substrate lattice parameter using density functional theory (DFT). First, DFT requires ordered SVON structures for input. We approximate the ordered structures for SVON-111 and SVON-112 using the OrderDisorderedStructureTransformations method within the *pymatgen* code.[8] The ordered cif files for the SVON-111 and SVON-112 compounds are provided in the Supplementary Files. From the ordering transformation, the resulting stoichiometry for SVON-111 is $Sr_5V_5O_{11}N_3$, and for ordered SVON-112 is $Sr_7V_7O_{14}N_5$. Note that the structural model for SVON-111 is the same as that used in Supplementary Note 2. Since the exact nitrogen content and its distribution in the SVON-112 is not clear, the structural model was constructed with reference to the SVON-111 structure; the isolated tetrahedral site is completely occupied by oxygen ($g_N = 0$ for the O7 site of the 7M structure shown in Supplementary Table 5), while the other sites are partially occupied by nitrogen atoms ($g_N = 1/5$ for the O1 and O3 sites, $g_N = 1/4$ for the O2, O4 and O5 sites, and $g_N = 1/2$ for the O6 site), giving a composition of $Sr_7V_7O_{14}N_5$.

The relative free-energies of the competing SVON phases with different stoichiometry can be compared using a thermodynamic potential with natural variables of ϵ_{2D}, biaxial strain; μ_O, the chemical potential of oxygen; and μ_N, the chemical potential of hydrogen.[9] ϵ_{2D} is an extensive variable, and so it is already a natural variable of *G*. The relevant potential can thus be constructed with a Legendre Transform as:

$$\Phi_{SVO_xN_y}\left(\ _{2D},\ \mu_O,\ \mu_N\right) = G_{SVO_xN_y} - \mu_O x_O - \mu_N x_N$$

In this work, the SVON phases are prepared by ammonolysis of $SrVO_3$ with flowing $NH_3(g)$ at 200 mL/min at 600 °C. A microkinetic model can be made from the following coupled processes:

$NH_3(g)$ → N(SVON lattice) + 1.5 $H_2(g)$

O(SVON lattice) + $H_2(g)$ → H_2O (g)

To compute the thermodynamic competition for the two orientations of SVO_xN_y, we therefore need to calculate $\mu_{N(lattice)}, \mu_{H_2(g)}, \mu_{O(lattice)}$ and $\mu_{H_2O(g)}$.

Katsura previously conducted a thorough thermodynamic analysis of $\mu_{N(lattice)}$ and $\mu_{H2(g)}$ in the nitridation of a metal using ammonia gas.[10] It was found that both μ_H and μ_N is significantly higher in flowing ammonia gas than would be expected from the direct equilibrium of $NH_3(g)$ → 1/2 $N_2(g)$ + 3/2 $H_2(g)$. This is due to the fact that NH_3 dissociation does not proceed to completion. The activity of nitrogen and hydrogen was found to depend on the NH_3 dissociation constant, which varies with ammonia gas flow rate; as well as with temperature. In a following paper, Katsura benchmarked on a

U_2N_{3+x} system that NH_3(g) has a dissociation constant of 0.3 at 600 °C for a flow rate of 50 mL/min.[11] However, a higher NH_3(g) flow rate was discussed to have a lower dissociation constant, with corresponding higher nitrogen activity.[12]

The experiments here are carried out at 600 °C and a flow rate of 200 mL/min, with the corresponding nitrogen activity and dissociation constant shown on Supplementary Fig. 9 below (as reproduced from Ref. [8]). We do not have the data to specify the exact NH_3 dissociation constant, but we know that the dissociation constant should be < 0.3, so we estimate the activity of nitrogen and hydrogen as $a_N = 10^4$, $a_H = 10^{1.3}$.

Because the oxygen and nitrogen stoichiometries between SVON-111 and SVON-112 are slightly different, errors in the nitrogen and hydrogen chemical potential could plausibly influence the equilibrium relationships between these two anion-vacancy orderings as a function of strain. In Supplementary Fig. 9, we also include error bars on the free-energy diagram corresponding to a range of nitrogen activities between $\log(a_N) = 4.8$ and $\log(a_N) = 3.3$. The figure shows that the equilibrium relationships between the two SVON anion-vacancy orderings as a function of strain are not very sensitive to potential errors in the nitrogen activity. The influence of hydrogen activity varying between $\log(a_H) = 1 - 1.5$ has negligible influence and the error bars are convolved with the nitrogen activity (since the relevant molecular specie is ammonia).

Next we assume that the activity of water has a negligible contribution to the chemical potential of water (in other words, that the water chemical potential is dominated by the *TS* term), so that $\mu_{H_2O} \sim \mu^o_{H_2O}$. The chemical potentials for oxygen and nitrogen in the SVON grand potential can therefore specified as:

$$\mu_N = \tfrac{1}{2}\mu^o_{N_2} + RT\ ln\ [a_N]$$

$$\mu_O = \mu^o_{H_2O} - 2(0.5\,\mu^o_{H_2} + RT\ln[a_H])$$

We calculate the standard state energies for $\mu^o_{H_2}$, $\mu^o_{H_2O}$, $\mu^o_{N_2}$ and $\mu^o_{O_2}$ in DFT under the SCAN metaGGA functional[13] using the molecule in a 10 Å × 10 Å × 10 Å box. To compare substrate lattice parameters, we relax perovskite $LaAlO_3$ and $SrTiO_3$ in DFT-SCAN as well. The A-A cation distance in the perovskite is $d_{La-La} = 5.390$ Å in $LaAlO_3$ and $d_{Sr-Sr} = 5.579$ Å in $SrTiO_3$. The corresponding lattice parameter of SVON-111 and SVON-112 is calculated by the nearest-neighbor Sr–Sr distance. This results in Figure 4g and Supplementary Figure 10 in the manuscript.

Supplementary Figures

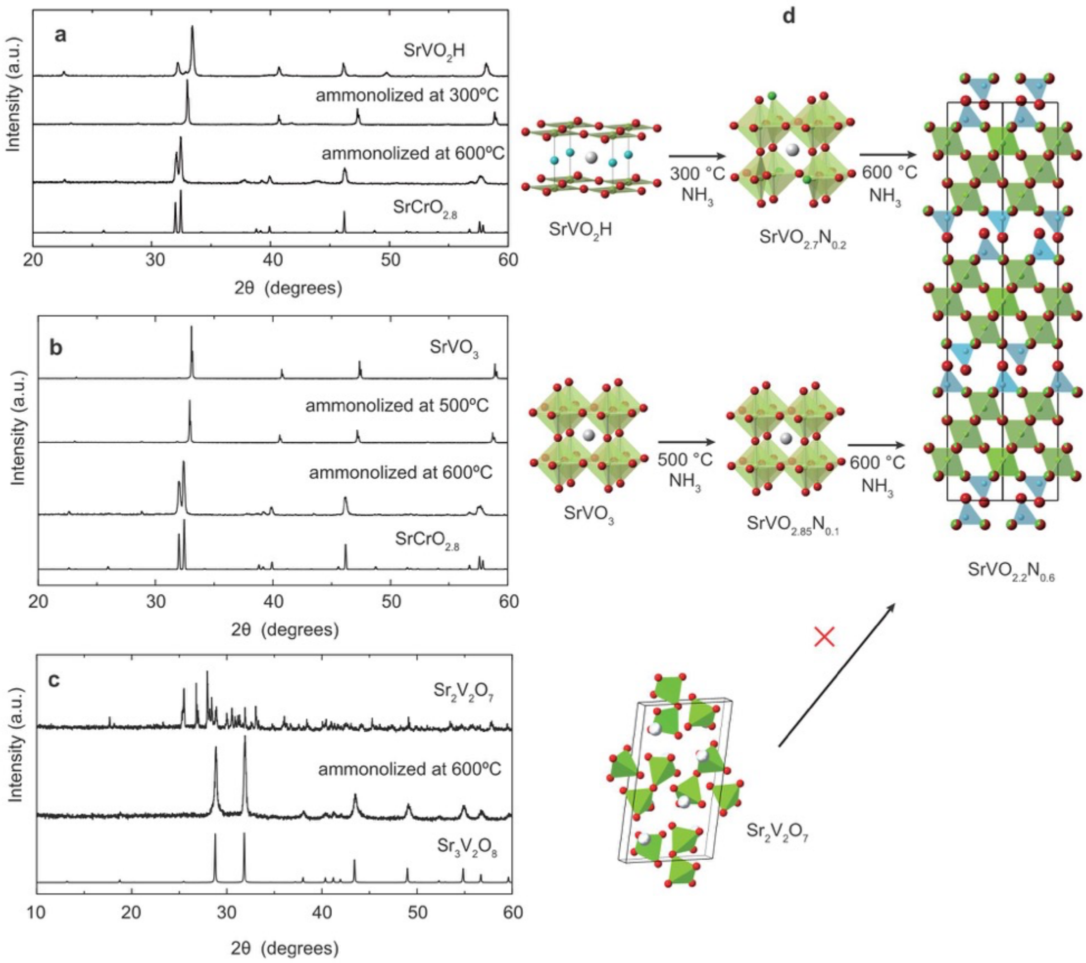

Supplementary Figure 1. Ammonolysis reactions of perovskite vanadates. a, XRD patterns of $SrVO_2H$ before and after the ammonolysis treatment at 300 °C and 600 °C. The former (300 °C) is a cubic perovskite $SrVO_{2.7}N_{0.2}$, while the latter (600 °C) has a *R*–3*m* structure. A simulation pattern of 15R-type $SrCrO_{2.8}$ is shown for comparison.[4] **b,** XRD patterns of $SrVO_3$ and the sample ammonolized at 500 °C and 600 °C. The former (500 °C) is a cubic perovskite $SrVO_{2.85}N_{0.1}$. c, XRD patterns of $Sr_2V_2O_7$ and the sample ammonolized at 600 °C. A simulation pattern of $Sr_3V_2O_8$ is shown.[5] **d,** Schematic view of ammonolysis reactions for $SrVO_2H$, $SrVO_3$, and $Sr_2V_2O_7$. White, red, and green spheres represent Sr, O, and N atoms, respectively. V atoms lie in the center of the polyhedra. Sr is omitted in the structure of $SrVO_{2.2}N_{0.6}$.

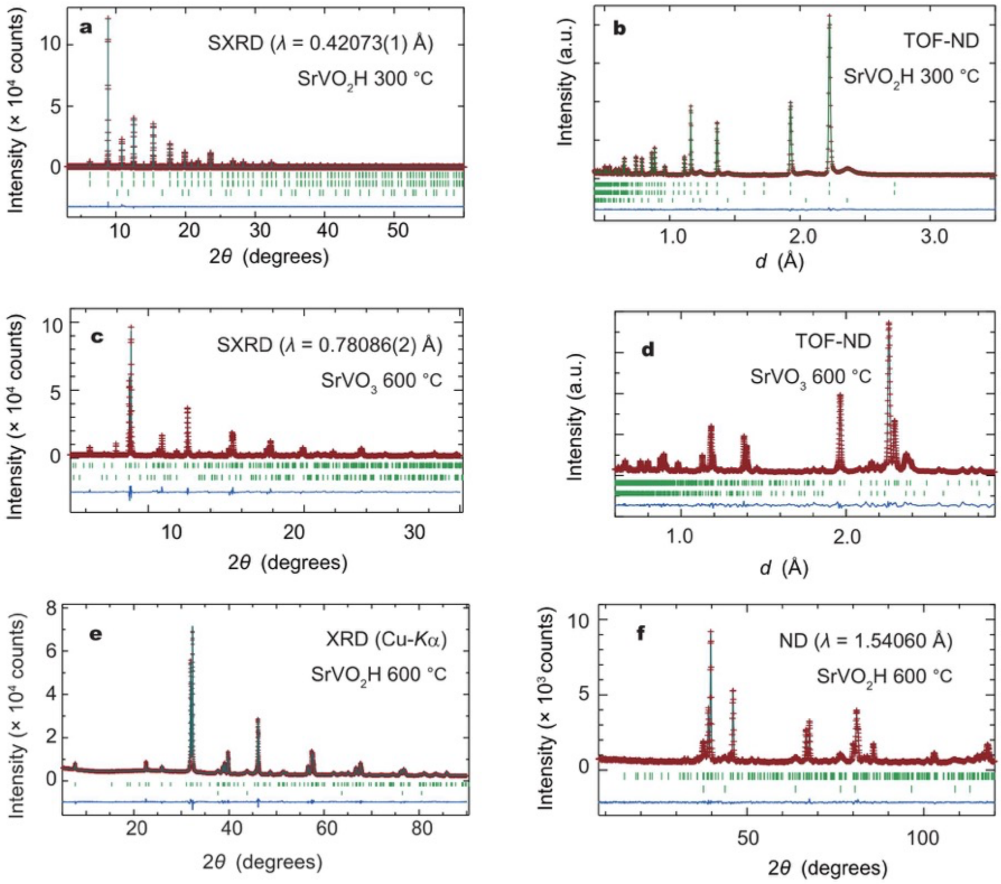

Supplementary Figure 2. Rietveld refinements of ammonolized samples. a, b, Room temperature synchrotron XRD (λ = 0.42073(1) Å at BL02B2, SPring-8) and neutron diffraction (ND) (Time of flight neutron at NOVA, J-PARC) patterns for the $SrVO_2H$ sample after ammonolysis at 300 °C ($SrVO_{2.7}N_{0.2}$). Red overlying crosses and green solid curves represent the observed and the calculated intensities, respectively. The blue solid lines at the bottom indicate the residual curves. The upper, middle, and lower green ticks indicate the peak positions of $SrVO_{2.7}N_{0.2}$, $SrVO_3$ (a mass fraction of 5%) and VN (a mass fraction of 7%). **c, d,** Room temperature synchrotron XRD (λ = 0.78086(2) Å at BL02B2) and neutron diffraction (at NOVA) patterns for the $SrVO_3$ sample after ammonolysis at 600 °C ($SrVO_{2.2}N_{0.6}$). The upper and lower green ticks indicate the peak positions of $SrVO_{2.2}N_{0.6}$ and $Sr_3V_2O_8$ (a mass fraction of 16%). **e, f,** Room temperature laboratory XRD (Cu-K_α radiation) and neutron diffraction (λ = 1.54060 Å at BT1, NIST) patterns for the $SrVO_2H$ sample after ammonolysis at 600 °C ($SrVO_{2.2}N_{0.6}$). The upper and lower green ticks indicate the peak positions of $SrVO_{2.2}N_{0.6}$ and VN (a mass fraction of 16%).

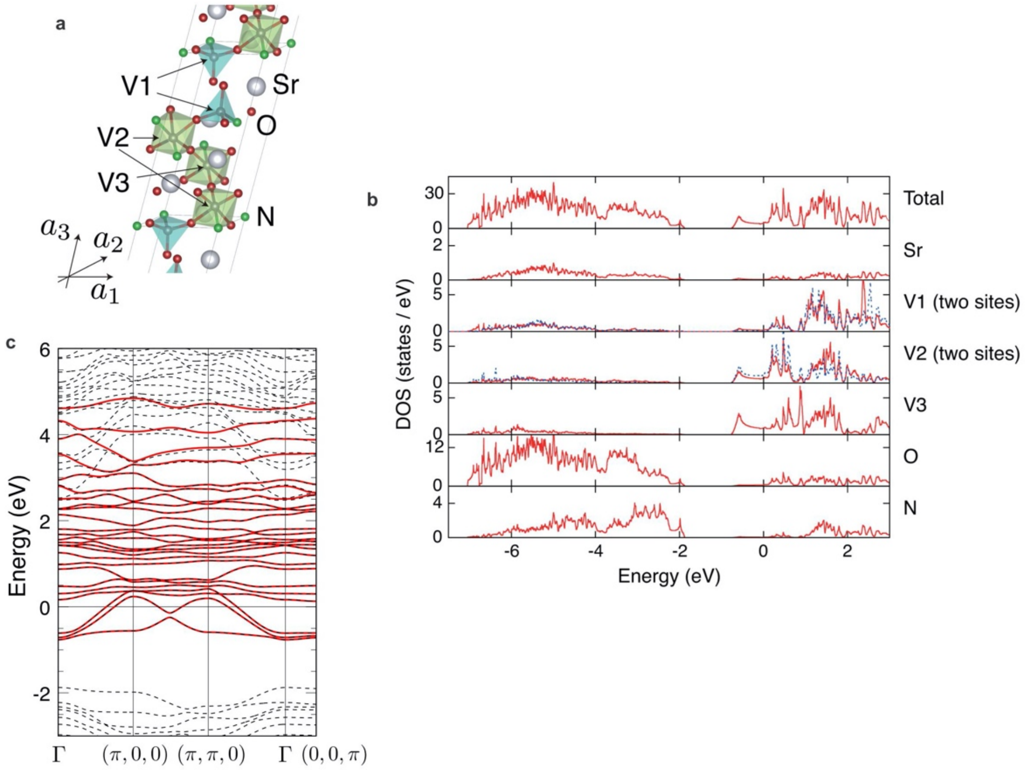

Supplementary Figure 3. First-principles calculation of $Sr_5V_5O_{11}N_3$ ($SrVO_{2.2}N_{0.6}$). a, Optimized crystal structure of $Sr_5V_5O_{11}N_3$. The unit cell vectors used in the band-structure calculation by the WIEN2k code are shown. b, Partial density of states (PDOS) for $Sr_5V_5O_{11}N_3$. Fermi energy is set to zero. Two lines for the V1 and V2 sites arise from the nitrogen substitution. **c,** First-principles band structure (black broken lines) and that calculated by the tight-binding model consisting of the Wannier functions (red solid lines) for $Sr_5V_5O_{11}N_3$.

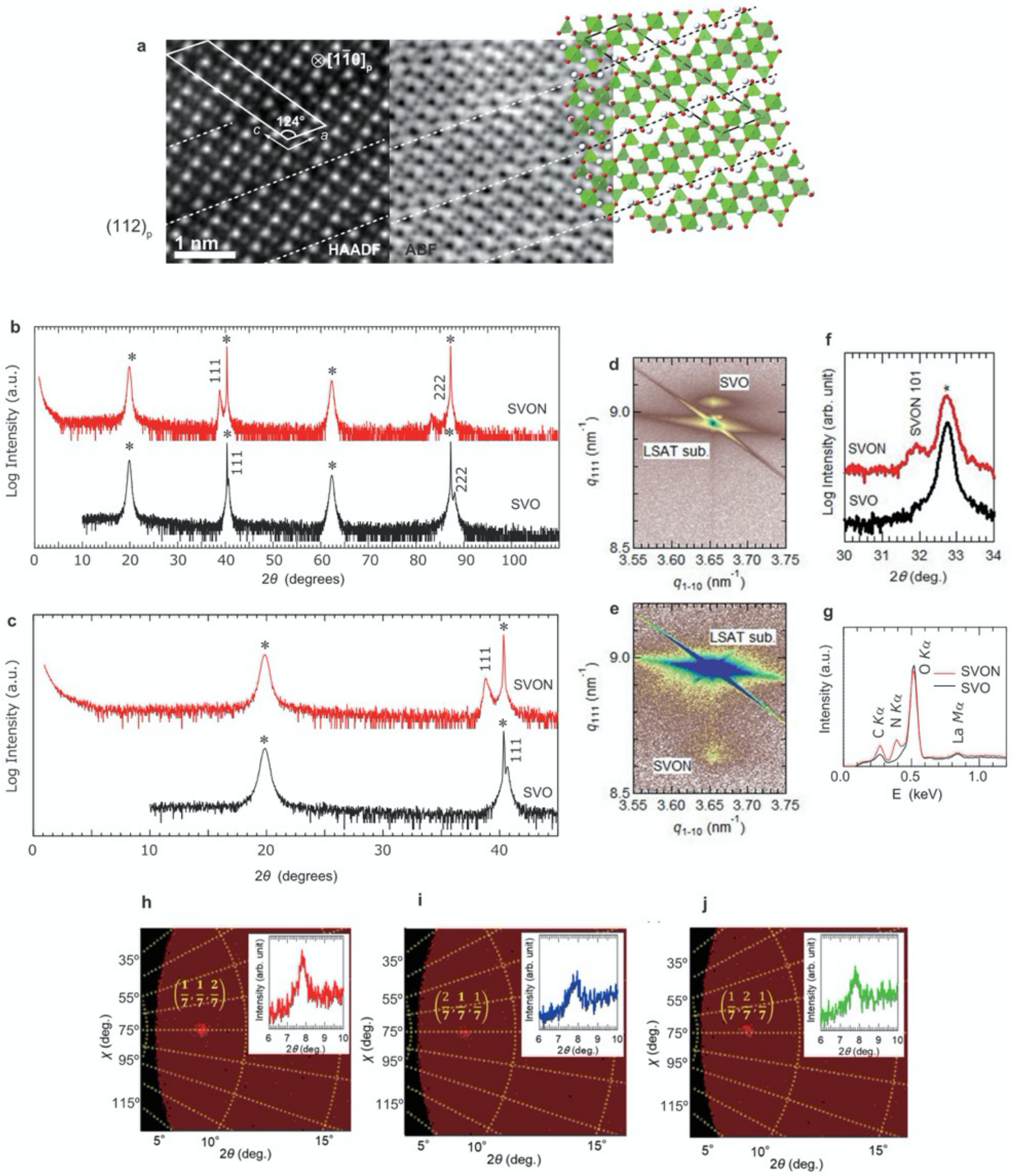

Supplementary Figure 4. Characterization of the ammonolized film on LSAT (111) substrate. a, High-resolution HAADF and ABF images of the film after ammonolysis (SVON). White frame in HAADF image represents the unit cell of the SVON. 7M-SrV(O,N)$_{2.71}$ structure (a = 7.11 Å, b = 5.48 Å, c = 27.0 Å, β = 124°). **b, c,** Out-of-plane XRD patterns of the as-deposit $SrVO_3$ film on LSAT (SVO) and the SVON film at 0°–110° (**b**) and 0°–45° (**c**). Asterisks represent peaks from LSAT substrate. No superlattice peak was detected in the SVON film. **d, e,** XRD reciprocal space maps of SVO (**d**) and SVON (**e**), measured around the LSAT 312 asymmetric diffraction. The q_{1-10} value of the oxynitride film was the same as that of the LSAT substrate, indicating that the epitaxial relationship

was retained after ammonolysis treatment. **f,** One-dimensional XRD pattern of $(101)_p$. g, EDS results of SVO and SVON films. **h-j,** Two-dimensional detector images of $(1/7\ 1/7\ 2/7)_p$ (**h**), $(2/7\ 1/7\ 1/7)_p$ (**i**) and $(1/7\ 2/7\ 1/7)_p$ (**j**) peaks in SVON/LSAT (111) film. Inset shows the 2θ–θ XRD patterns obtained by the integration along χ direction. The presence of equivalent three peaks suggests the existence of the three types of domains rotated by 120° with respect to each other.

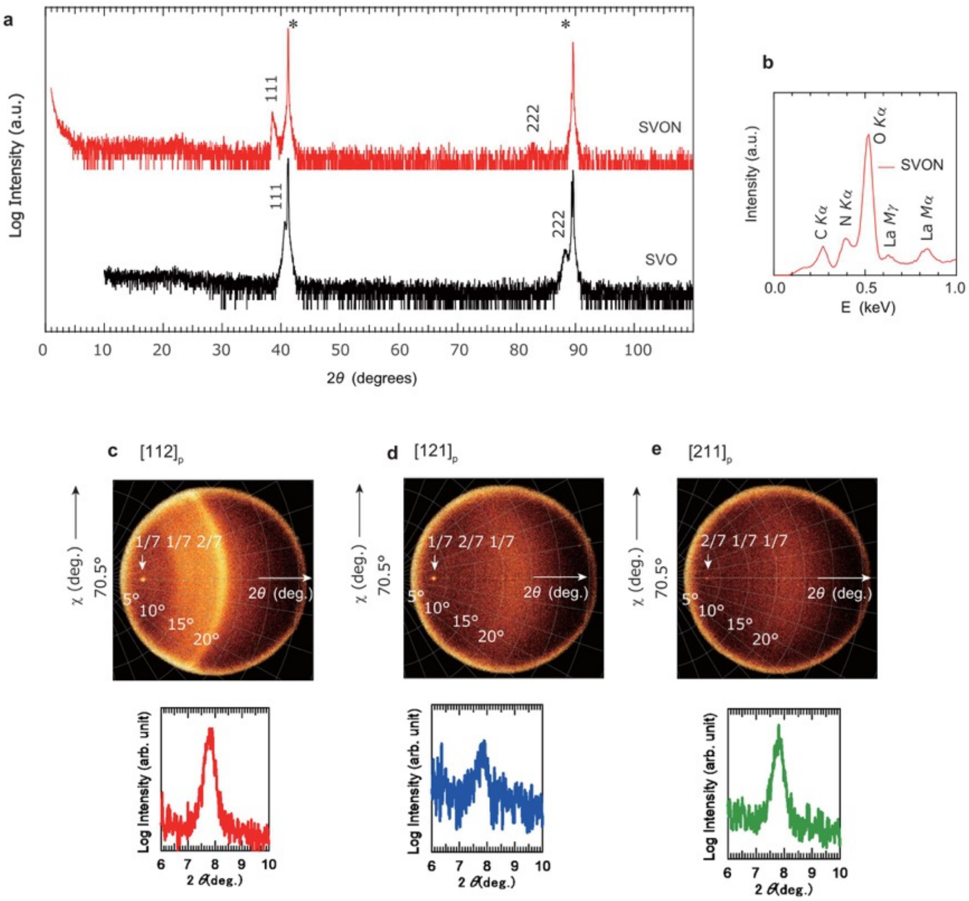

Supplementary Figure 5. Characterization of the ammonolized SVO_3 film on $LaAlO_3$ (111) substrate. a, Out-of-plane XRD patterns of the as-deposit film on $LaAlO_3$ (SVO) and the film after ammonolysis (SVON) at 0°~110°. b, EDS results of the SVON film. **c-e,** Two-dimensional detector images of (1/7 1/7 2/7) (**c**), (2/7 1/7 1/7) (**d**) and (1/7 2/7 1/7) (**e**) peaks in SVON/$LaAlO_3$ (111) film. Lower profile shows the 2θ–θ XRD patterns obtained by the integration along χ direction. The out-of-plane distance of 2.33 Å (cf. 2.32 Å for the film on LSAT) determined by the 111 peak in a suggests that the film grown on $LaAlO_3$ substrate was partially relaxed to around −1.0% because of too large lattice mismatch (−2.3%).

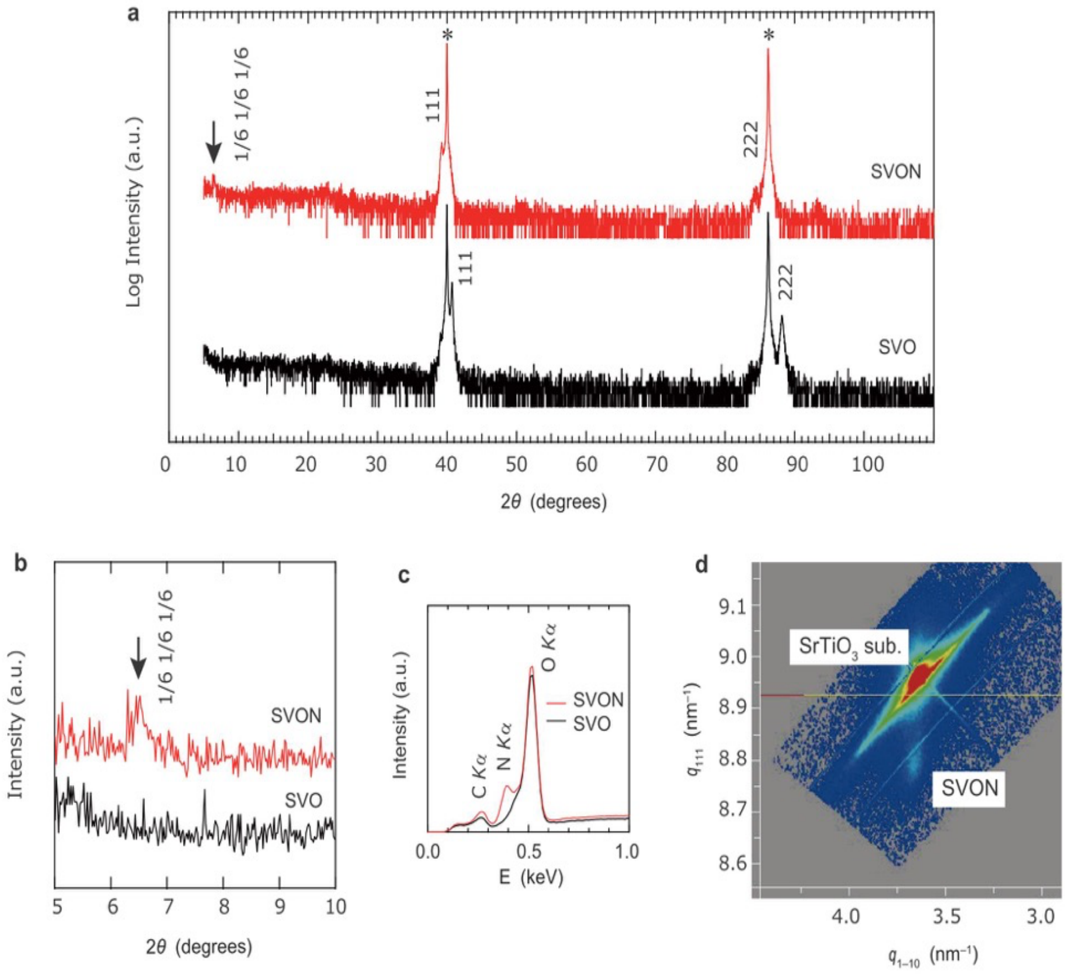

Supplementary Figure 6. Characterization of the ammonolized film on $SrTiO_3$ (111) substrate. a, b, Out-of-plane XRD patterns of the as-deposit film on $SrTiO_3$ (SVO) and the film after ammonolysis (SVON) at 0°–110° (**a**) and 5°–10° (**b**). Asterisks represent peaks from the substrate. The (1/6 1/6 1/6) superlattice peak was detected at 6.5° in the SVON film (red). **c,** EDS results of SVO (black) and SVON (red) films. **d,** XRD reciprocal space maps of SVON, measured around the $SrTiO_3$ 312 asymmetric diffraction. The q_{1-10} value of the oxynitride film was the same as that of the $SrTiO_3$ substrate, indicating that the epitaxial relationship was retained after ammonolysis.

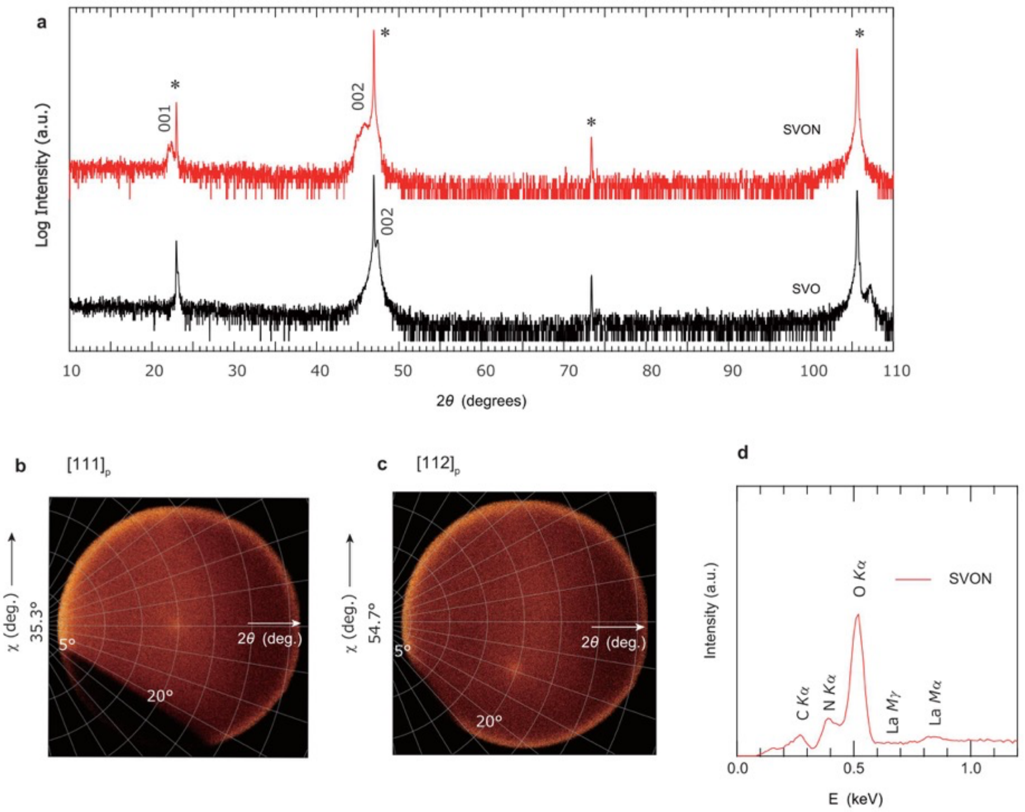

Supplementary Figure 7. Characterization of the ammonolized film on LSAT (100) substrate. a, Out-of-plane XRD patterns of the as-deposit film on LSAT (SVO) and the film after ammonolysis (SVON) at 0°–110°. Asterisks represent peaks from the substrate. **b, c,** Two-dimensional detector images of SVON/LSAT (100) film along [111] (**b**) and [112] (**c**), where no reflection peak can be detected. **c,** EDS results of the SVON film. **d,** EDS results of the SVON film.

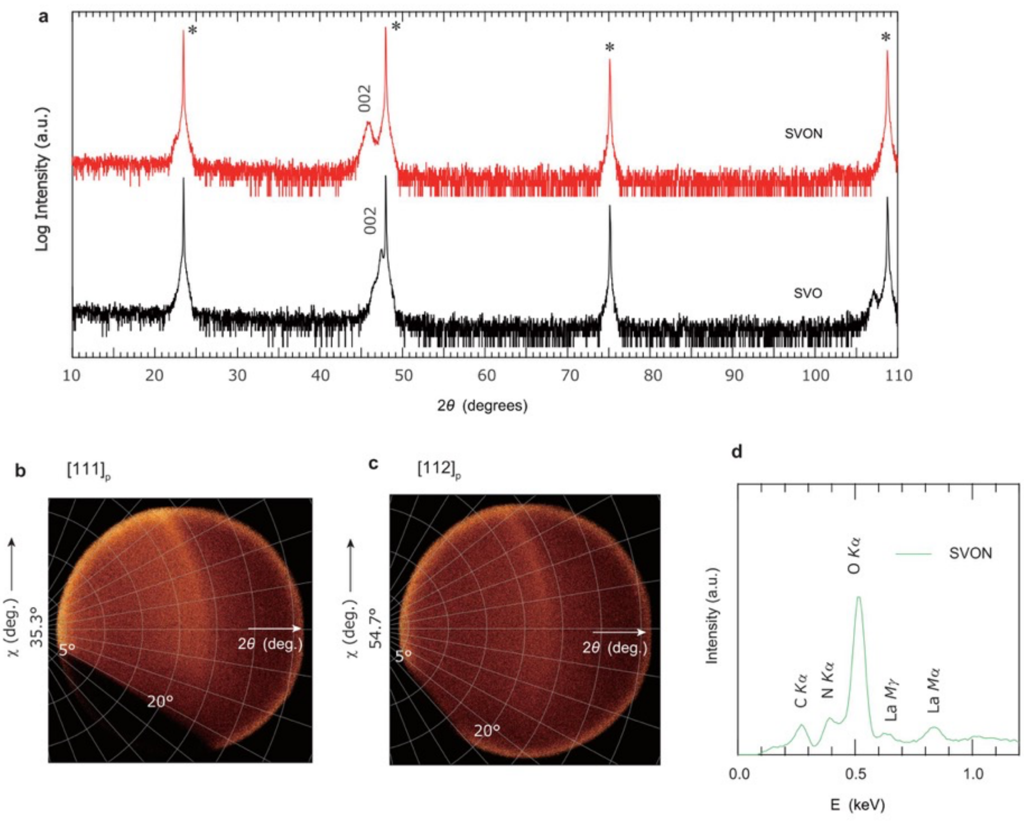

Supplementary Figure 8. Characterization of the ammonolized $SrVO_3$ film on $LaAlO_3$ (100) substrate. a, Out-of-plane XRD patterns of the as-deposit film on $LaAlO_3$ (SVO) and the film after ammonolysis (SVON) at 0°–110°. Asterisks represent peaks from the substrate. **b, c,** Two-dimensional detector images of SVON/LSAT (100) film along [111] (**b**) and [112] (**c**), where no reflection peak can be detected. **d,** EDS results of the SVON film.

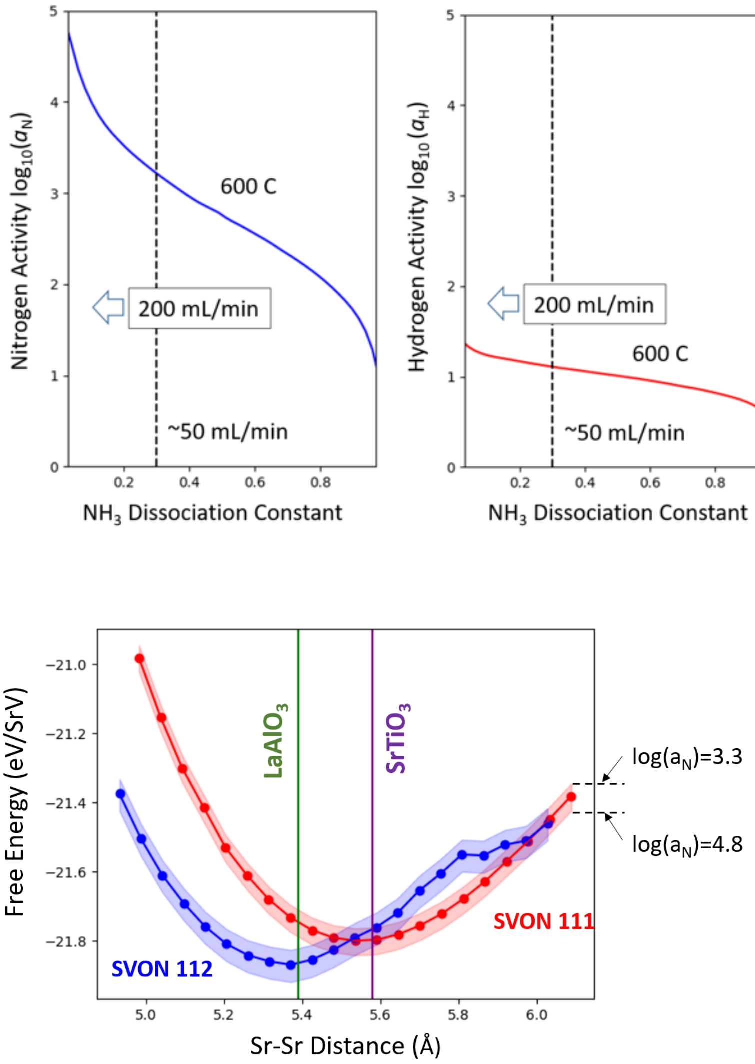

Supplementary Figure 9. Activities of nitrogen and oxygen as a function of NH_3 dissociation constant. Our NH_3 flow rate (200 mL/min) is higher than in a previous benchmark (50 mL/min),[10] which had a dissociation constant of 0.3. Therefore, our dissociation constant should be lower than 0.3. Below, we include error bars on the free-energy diagram corresponding to a range of nitrogen activities between $\log(a_N) = 4.8$ and $\log(a_N) = 3.3$. Variations in the oxygen and nitrogen stoichiometry between SVON-111 and 112 do not significantly affect the equilibrium relationships between the two anion-vacancy orderings as a function of strain.

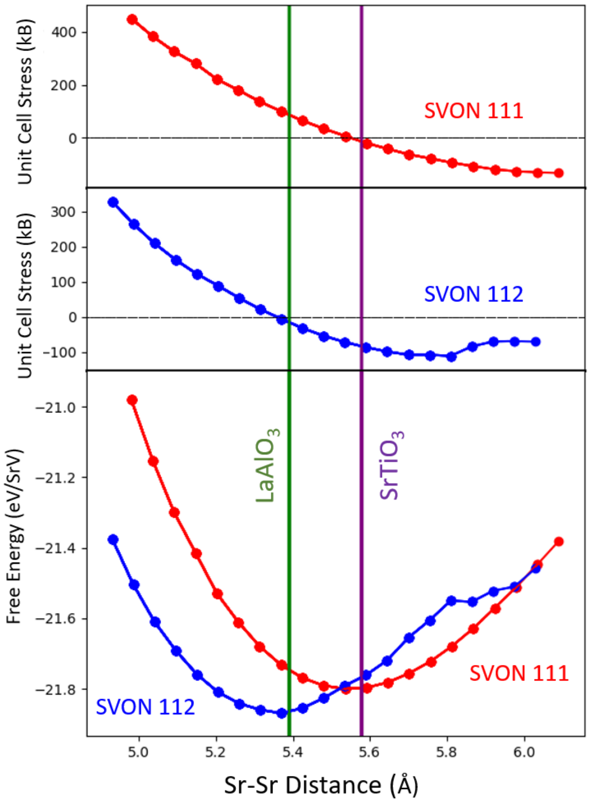

Supplementary Figure 10. Unit cell stresses in the SVON-111 and SVON-112 structures as a function of the Sr–Sr distance, as referenced against Figure 4g. The SVON unit cells are strained isotropically in the 2D biaxial directions.

Supplementary Tables

Supplementary Table 1. Crystallographic parameters for $SrVO_{2.7}N_{0.2}$ ammonolized from $SrVO_2H$ at 300 °C, obtained from SXRD (upper) and ND (lower) refinements.[a]

atom	site	*g*	*x*	*y*	*z*	U_{iso} / ×100 Å^2
Sr	1*a*	1	0.5	0.5	0.5	0.62(1)
						0.37(4)
V	1*b*	1	0	0	0	0.46(2)
O	3*c*	0.976(5)	0.5	0	0	0.55(4)
O/N		0.90(1)/0.07(1)				0.53(4)

[a] Space group: *Pm*–3*m*; *Z* = 1; *a* = 3.84492(3) Å for SXRD and *a* = 3.85009(3) Å for ND. R_p = 5.24%, R_{wp} = 7.27%, R_B = 4.99% for SXRD and R_p = 2.88%, R_{wp} = 3.21%, R_B = 0.83% for ND.

Supplementary Table 2. Crystallographic parameters for $SrVO_{2.2}N_{0.6}$ ammonolized from $SrVO_3$ at 600 °C, obtained from SXRD (upper) and ND (lower) refinements.[a]

atom	site	*g*	*x*	*y*	*z*	U_{iso} / ×100 Å^2
Sr1	3*a*	1	0	0	0	1.23(7)
						1.78(3)
Sr2	6*c*	1	0	0	0.4056(2)	1.23(7)
					0.40734(4)	1.78(3)
Sr3	6*c*	1	0	0	0.1987(2)	1.23(7)
					0.19715(5)	1.78(3)
V1	6*c*	1	0	0	0.2900(3)	0.6(1)
V2	6*c*	1	0	0	0.0992(2)	0.6(1)
V3	3*b*	1	0	0	0.5	0.6(1)
O1	6*c*	1	0	0	0.3274(8)	1.27
					0.38517(7)	4.85(7)
O2/N2	18*h*	1/0	0.501(3)	0.499(3)	0.3996(7)	1.27
		0.675(8)/0.325(8)	0.4954(1)	0.5046(1)	0.39905(2)	1.20(2)
O3/N3	18*h*	1/0	0.499(3)	0.501(3)	0.2029(7)	1.27
		0.827(5)/0.173(5)	0.4993(1)	0.5008(1)	0.20074(2)	0.12(2)

[a] Space group: *R*–3*m*; *Z* = 15; *a* = 5.5305(2) Å, *c* = 34.4874 (9) Å for SXRD and *a* = 5.52234(1) Å, *c* = 34.3888 (9) Å for ND. R_p = 8.29%, R_{wp} = 10.76%, R_B = 5.09% for SXRD and R_p = 7.72%, R_{wp} = 11.3%, R_B = 4.77% for ND.

Supplementary Table 3. Crystallographic parameters for $SrVO_{2.2}N_{0.6}$ ammonolized from $SrVO_2H$ at 600 °C, obtained from SXRD (upper) and ND (lower) refinements.[a]

atom	site	*g*	*x*	*y*	*z*	U_{iso} / ×100 Å^2
Sr1	3*a*	1	0	0	0	1.14(4)
						0.5(2)
Sr2	6*c*	1	0	0	0.40567(7)	1.14(4)
					0.4069(1)	0.7(2)
Sr3	6*c*	1	0	0	0.19627(8)	1.14(4)
					0.1960(2)	0.3(2)
V1	6*c*	1	0	0	0.29016(9)	0.39(5)
V2	6*c*	1	0	0	0.099(1)	0.39(5)
V3	3*b*	1	0	0	0.5	0.39(5)
O1	6*c*	1	0	0	0.3343(2)	0.29(8)
					0.3382(3)	[b]
O2/N2	18*h*	1/0	0.5	0.5	0.3943(2)	0.29(8)
		0.66(3)/0.34(3)	0.4999(8)	0.5001(8)	0.39746(0)	1.5(1)
O3/N3	18*h*	1/0	0.5	0.5	0.2009(2)	0.29(8)
		0.85(3)/0.15(3)	0.4994(8)	0.5007(8)	0.2000(1)	0.9(1)

[a] Space group: *R*–3*m*; $Z = 15$; $a = 5.53412(7)$ Å, $c = 34.5250(5)$ Å for XRD and $a = 5.5354(2)$ Å, $c = 34.535(1)$ Å for ND. $R_p = 2.48\%$, $R_{wp} = 3.37\%$, $R_B = 1.41\%$, GoF = 2.16 for XRD and $R_p = 3.34\%$, $R_{wp} = 4.29\%$, $R_B = 2.19\%$, GoF = 1.18 for ND. Values in parentheses indicate one standard deviation. [b] $U_{11} = U_{22} = 2 \times U_{12} = 0.153(7)$ Å^2, $U_{33} = 0.003(5)$ Å^2, $U_{13} = U_{23} = 0$.

Supplementary Table 4. **Bond lengths of 15R-$SrVO_{2.2}N_{0.6}$.**

Bond	Number of the bonds	Bond length (Å)	Bond valence sum[a]
V1–O1	1	1.656	+5.1 for V1
V1–O2/N2	3	1.754	
V2–O2/N2	3	2.001	+3.6 for V2
V2–O3/N3	3	1.995	
V3–O3/N3	6	1.964	+3.8 for V3
Sr1–O1	6	3.2002	+2.2 for Sr1
Sr1–O2/N2	6	2.731	
Sr2–O1	1	2.37	+2.3 for Sr2
Sr2–O2/N2	6	2.771	
Sr2–O3/N3	3	2.606	
Sr3–O2/N2	6	2.991	+2.0 for Sr3
Sr3–O3/N3	6	2.771	

[a] Bond valence sum values[14, 15] are calculated by assuming average coordination geometries.

Supplementary Table 5. Crystallographic parameters for the 7M structure.[a]

atom	multiplicity	*x*	*y*	*z*
Sr1	2	0.5	0	0
Sr2	4	0.515	0	0.43
Sr3	4	0.48	0	0.141057
Sr4	4	0.552	0	0.286
V1	2	0	0	0
V2	4	0.97	0	0.426
V3	4	0.09	0	0.135
V4	4	0	0	0.305
O1	8	0.25	0.25	0.035714
O2	4	0	0	0.071429
O3	2	0	0	0.5
O4	4	0	0	0.3538
O5	8	0.25	0.25	0.607143
O6	8	0.225	0.25	0.185
O7	4	0.65	0	0.266

[a] $SrVO_{2.71}$ ($Sr_{14}V_{14}O_{38}$). Space group: *I*2/*m* (No. 8); $Z = 14$; $a = 7.11$ Å, $b = 5.48$ Å, $c = 27.0$ Å, $\beta = 124°$. Note that *C*/*m* is the standard setting of this space group. We used *I*2/*m* as its [001] is identical to $[001]_p$ for the cubic perovskite.

Supplementary Table 6. Bond lengths of 7M-$SrVO_{2.71}$ ($Sr_{14}V_{14}O_{38}$).

Bond	Number of the bonds	Bond length (Å)	Bond valence sum [a]
V1–O1	4	2.012	+3.51 for V1
V1–O2	2	1.929	
V2–O1	2	2.012	+3.35 for V2
V2–O3	1	1.998	
V2–O4	1	1.949	
V2–O5	2	2.015	
V3–O2	1	1.844	+3.30 for V3
V3–O5	2	1.89	
V3–O6	2	2.061	
V4–O4	1	1.757	+4.37 for V4
V4–O6	2	1.812	
V4–O7	1	1.711	
Sr1–O1	4	2.808	+1.68 for Sr1
Sr1–O3	2	2.74	
Sr1–O5	4	2.767	
Sr2–O1	2	2.87	+1.61 for Sr2
Sr2–O1	2	2.735	
Sr2–O2	2	2.742	
Sr2–O5	2	2.747	
Sr2–O6	2	2.916	
Sr3–O1	2	2.727	+1.63 for Sr3
Sr3–O4	2	2.743	
Sr3–O5	2	2.892	
Sr3–O6	2	2.996	
Sr3–O7	1	2.416	
Sr4–O4	2	2.641	+1.68 for Sr4
Sr4–O5	2	2.764	
Sr4–O6	2	2.762	
Sr4–O6	2	2.845	
Sr4–O7	2	2.951	

[a] Bond valence sum[14, 15] was calculated by placing O^{2-} ions for all anion sites. Thus, the obtained value should be more or less underestimated. BVS of +3.3~3.5 are obtained for the octahedral (V1, V2) and pyramidal (V3) sites, while +4.4 is obtained for the tetrahedral (V4) site.

Supplementary references

1. Denis Romero, F. *et al*. Strontium vanadium oxide–hydrides: "square–planar" two–electron phases. *Angew. Chem. Int. Ed.* **53**, 7556-7559 (2014).

2. Yajima, T. *et al*. A labile hydride strategy for the synthesis of heavily nitridized $BaTiO_3$. *Nat. Chem.* **7**, 1017-1023 (2015).

3. Hosoya, S., Yamagishi, T. & Tokonami, M. Study of electron state in vanadium nitride by intensity measurements of X-ray diffraction. *J. Phys. Soc. Jpn.* **24**, 363-367 (1968).

4. Arévalo–López, A. M. *et al*. "Hard–soft" synthesis of $SrCrO_{3-\delta}$ superstructure phases. *Angew. Chem. Int. Ed.* **51**, 10791-10794 (2012).

5. Carrillo–Cabrera, W. & Von Schnering, H. G. Crystal structure refinement of strontium tetraoxo–vanadate (V), $Sr_3(VO_4)_2$. *Z. Kristal.* **205**, 271-278 (1993).

6. Oró–Solé, J., Clark, L., Bonin, W., Attfield, J. P. & Fuertes, A. Anion–ordered chains in a d^1 perovskite oxynitride: $NdVO_2N$. *Chem. Commun.* **49**, 2430-2432 (2013).

7. Kawamura, M. FermiSurfer: Fermi-surface viewer providing multiple representation schemes. *Comput. Phys. Commun.* **239**, 197-203 (2019).

8. Ong, S. P. *et al*. Python Materials Genomics (pymatgen): A robust, open-source python library for materials analysis. *Comput. Mater. Sci.* **68**, 314-319 (2013).

9. Alberty, R. A. Use of Legendre transforms in chemical thermodynamics (IUPAC Technical Report). *Pure and App. Chem.* **73**, 1349-1380 (2001).

10. Katsura, M. Thermodynamics of nitride and hydride formation by the reaction of metals with flowing NH_3. *J. Alloys Compounds* **182**, 91-102 (1992).

11. Katsura, M. & Serizawa, H. Formation of a nitrogen-rich α-U_2N_{3+x} phase by the reaction of uranium with a stream of ammonia. *J. Alloys Compounds* **187**, 389-399 (1992).

12. Katsura, M. A thermodynamic analysis of nitrogen-rich uranium sesquinitride formation by the reaction of uranium with ammonia. *Solid State Ionics* **49**, 225-231 (1991).

13. Sun, J., Ruzsinszky, A. & Perdew, J. P. Strongly constrained and appropriately normed semilocal density functional. *Phys. Rev. Lett.* **115**, 036402 (2015).

14. Brese, N. & O'keeffe, M. Bond–valence parameters for solids. *Acta Cryst.* **B47**, 192-197 (1991).

15. Brown, I. D. & Altermatt, D. Bond-valence parameters obtained from a systematic analysis of the inorganic crystal structure database. *Acta Cryst.* **B41**, 244-247 (1985).
